# APPA-3D: an autonomous 3D path planning algorithm for UAVs in unknown complex environments

**DOI:** 10.1038/s41598-024-51286-2

**Published:** 2024-01-12

**Authors:** Jintao Wang, Zuyi Zhao, Jiayi Qu, Xingguo Chen

**Affiliations:** 1 Civil Aviation College, Shenyang Aerospace University, Shenyang, 110136 China; 2 Henan Shijia Photons Technology Co., Ltd, Hebi, 458030 China

**Keywords:** Aerospace engineering, Computer science, Computational science

## Abstract

Due to their high flexibility, low cost, and ease of handling, Unmanned Aerial Vehicles (UAVs) are often used to perform difficult tasks in complex environments. Stable and reliable path planning capability is the fundamental demand for UAVs to accomplish their flight tasks. Most researches on UAV path planning are carried out under the premise of known environmental information, and it is difficult to safely reach the target position in the face of unknown environment. Thus, an autonomous collision-free path planning algorithm for UAVs in unknown complex environments (APPA-3D) is proposed. An anti-collision control strategy is designed using the UAV collision safety envelope, which relies on the UAV's environmental awareness capability to continuously interact with external environmental information. A dynamic reward function of reinforcement learning combined with the actual flight environment is designed and an optimized reinforcement learning action exploration strategy based on the action selection probability is proposed. Then, an improved RL algorithm is used to simulate the UAV flight process in unknown environment, and the algorithm is trained by interacting with the environment, which finally realizes autonomous collision-free path planning for UAVs. The comparative experimental results in the same environment show that APPA-3D can effectively guide the UAV to plan a safe and collision-free path from the starting point to the target point in an unknown complex 3D environment.

## Introduction

Unmanned Aerial Vehicles (UAVs) are widely used in a variety of scenarios due to their abilities of high flexibility, high productivity, ease of maneuverability, and adapting to hazardous environments. The increasing complexity of flight environments requires UAVs to have the ability to interact with highly dynamic and strongly real-time space operating environments, which put forward new demands for UAVs’ autonomy and safety. UAVs detect and determine whether there is a potential conflict in the future period through the sensors so that they can maintain a certain safe distance from the dynamic/static obstacles in the airspace, and thus plan an ideal flight path from the starting point to the target point and avoid conflicts.

**Figure 1:**
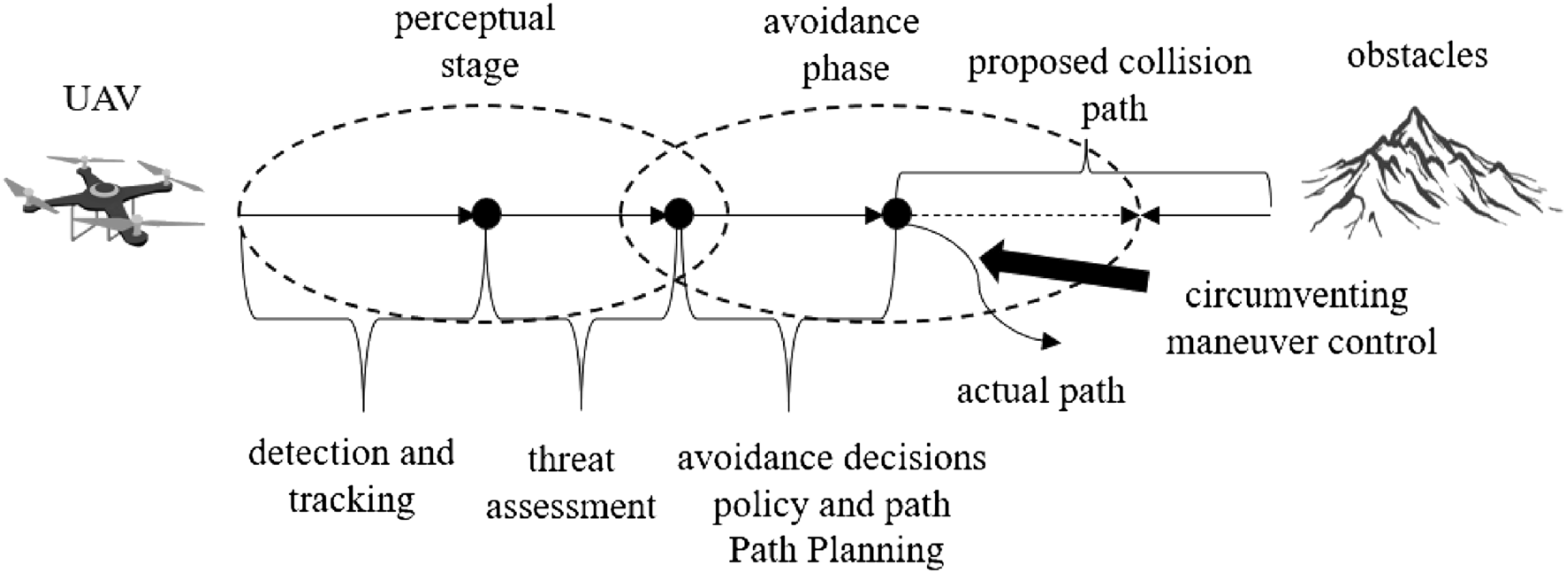
Schematic diagram of UAV perception and avoidance.

Unlike civil aircraft, UAVs usually perform tasks in lower airspace. There are many static obstacles in lower airspace such as buildings, trees, and dynamic aircraft. Flight conflict is a state when the distance between two aircraft in the direction of horizontal, longitudinal, or vertical is less than a specific interval resulting in the aircraft being at risk^1^. UAVs are required to have autonomous environment sensing, collision threat estimation, avoidance path planning, and maneuver control. These abilities are referred to as Sense And Avoid (SAA). Airspace environment sensing in UAV SAA refers to the detection and acquisition of various static/moving, cooperative/non-cooperative targets in the flying space, based on the onboard sensors or data links carried by the UAV, and evaluating the environmental situation and the degree of collision threat^2^. As shown in Fig. 1, SAA is an important safety guarantee for future UAV airspace integration applications and is also an important sign of autonomy and intelligence of UAVs^3^.

For UAVs, the ability of SAA is extremely important. The ability of path planning in the avoidance function of UAVs is an important foundation for the basis for them to complete the flight task. Complex flight environments put forward higher demands for path planning algorithms of UAVs, thus the research in autonomous obstacle avoidance path planning algorithms for UAVs is necessary.

It has to find the optimal flight path from the initial location to the target location under the constraints of environmental factors such as terrain, weather, threats, and flight performance of autonomous path planning for UAVs. Significantly, the optimal path does not always mean the shortest path or a straight line between two locations; instead, the UAV aims to find a safe path under limited power and flight task. There are a lot of UAV path planning algorithms, such as the Voronoi diagram algorithm, Rapidly-exploring Random Tree (RRT) algorithm, A* algorithm, etc. However, these algorithms cannot deal with dynamic environments effectively because they require global environmental information to calculate the optimal result. Once the environment changes, the original results will fail. Furthermore, the process of recalculating the optimal results is too slow for real-time operations because of the large number of calculations required. The above algorithms may still be effective if the obstacle is moving slowly. But when moving faster, the movement of the surrounding vehicles may cannot be predicted thus result in a collision. These shortcomings limit the application of the above algorithms to UAVs in real, dynamic environments.

To address these shortcomings, reinforcement learning algorithms are applied to the path planning process. Reinforcement learning (RL) is a branch of machine learning. UAVs can learn through continuous interaction with the environment, using training and learning to master the environment gradually, and optimize the state-behavior continuously to obtain the optimal strategy through the feedback (rewards) given by the environment, which is closer to the human learning process.

Compared with traditional algorithms, RL performs better when the environment is unknown and dynamic. Moreover, the inference speed and generalization of RL have advantages in real-time decision-making tasks. Therefore, the path planning algorithm based on RL has certain advantages in solving the UAV path planning problem in unknown and dynamic environments.

This paper considers the real-time and location limitation characteristics of path planning and refers to the existing research on UAV path planning problems and collision avoidance strategies for various stationary/motion threats. An autonomous collision-free path planning algorithm for UAVs in unknown complex 3D environments (APPA-3D) is proposed. Thus, UAVs can perform tasks with APPA-3D more safely and efficiently in complex flight environments. Firstly, the UAV spherical safety envelope is designed to research the anti-collision avoidance strategy, which will be used as an action plan for UAVs to realize dynamic obstacle avoidance. Secondly, we assume that the environment model when path planning is unknown, so the UAV needs to have the ability to learn and adjust flight state intelligently according to its surroundings. In this paper, the traditional model-free RL algorithm is improved to reduce the complexity of the algorithm and adapt to the demands of UAV path planning in an unknown complex 3D environment. It takes into account the search efficiency while guaranteeing the optimal search path.

Compared with the existing research, the innovative work of this paper mainly manifests in the following several aspects:

Based on the UAV environment sensing capability, a collision safety envelope is designed, and the anti-collision control strategy is studied concerning the Near Mid Air Collision (NMAC) rules for civil airliners and the International Regulations for Preventing Collisions at Sea (COLREGS). It provides a theoretical basis for UAVs to carry out collision detection and avoidance schemes, which can detect and avoid dynamic threats effectively in the flight environment.

To address the difficulty of convergence of traditional algorithms in solving 3D path planning. The artificial potential field method (APF) is used to optimize the mechanism of reward function generation in RL. The optimized algorithm can output the dynamic reward function by combining the actual flight environment information. Thus, the problems of path planning convergence difficulty, unreachable target point and model stop learning in high dimensional space caused by sparse reward function are solved.

Aiming at the "exploration-exploitation dilemma" of RL in the path planning process of UAVs, an RL action exploration strategy based on action selection probability is proposed. The strategy dynamically adjusts the action selection strategy by combining the size of the value function in different states, thus solving the RL exploration-exploitation problem and improving the efficiency of path search.

The rest of the paper is organized as follows: Section "Related research on UAV path planning" introduces the research status of UAV path planning; an anti-collision control strategy for UAVs is designed in Section "UAV anti-collision control strategy"; an Autonomous collision-free path planning algorithm is proposed in Section "Design of autonomous collision-free path planning algorithm for UAVs"; simulation experiment design and result analysis are presented in Section "Experiment and results"; the paper is summarized in Section "Conclusion".

## Related research on UAV path planning

Autonomous mobile robots (AMRs) has attracted more and more attention due to their practicality and potential uses in the modern world^4^. AMRs is widely used in different fields, such as agricultural production^5,6^, unmanned underwater vehicles (AUVs)^7,8^, automated guided vehicles (AGVs)^9^, autonomous cleaning robots^10^, industrial robots^11,12^, etc. The similarity of the above studies is that they are all need 3D path planning algorithms. Path planning is one of the most important tasks in AMR navigation since it demands the robot to identify the best route based on desired performance criteria such as safety margin, shortest time, and energy consumption. As an important part of AMRs, with the popularization of consumer-grade UAVs, the research on path planning of UAVs has become a hot topic.

UAV path planning refers to the formulation of the optimal flight path from the initial location to the target location, considering environmental factors such as terrain, meteorology, threats, and their flight performance constraints The aim is to improve the reliability and safety of UAVs while ensuring the efficiency of their task execution.

A lot of research has been done on the UAV path planning problem. Sampling-based path planning algorithms are widely used in UAV path planning due to their simplicity, intuitiveness, and ease of implementation. A simple sampling-based path planning algorithm is the Voronoi diagram algorithm^13^. The Voronoi diagram algorithm transforms the complex problem of searching for a trajectory in a spatial region into a simple search problem with a weighted diagram. However, the Voronoi diagram algorithm is only suitable for solving 2D path planning problems. 2D path planning divides the flight environment into passable and impassable areas through "rasterization" processing, and then route planning is performed on the processed map. The algorithm is easy to implement and is more intuitive and feasible, but it is difficult to consider terrain following, terrain avoidance, and threat avoidance simultaneously. Therefore, it is necessary to consider the real sense of 3D route planning with real-time and effective requirements to solve the UAV path planning problem in real scenarios. Another intuitive algorithm is the Rapidly exploring Random Tree (RRT)^14^. RRT can quickly and efficiently search in the smallest possible space, avoiding the need to model the space, and can effectively solve motion planning problems with high-dimensional spaces and complex constraints. However, it is less repeatable and the planned paths are often far from the shortest path.

In node-based path planning algorithms, Dijkstra's algorithm searches for the shortest path by cyclic traversal^15^, However, as the complexity of the flight map increases and the number of nodes increases, Dijkstra's algorithm suffers from too low execution efficiency. Reference^16^ designed the solution model of the "Dijkstra -based route planning method", which simplifies the search path, reduces the calculation amount, and improves the execution efficiency through the optimization of correction strategies, correction schemes, and O-D Adjacency matrix processing methods, thereby improving the traditional Dijkstra algorithm. The A* algorithm is a classical and commonly used heuristic search algorithm^17^. The A* algorithm guides the search through heuristic information to achieve the purpose of reducing the search range and improving the computational speed and can obtain real-time feasible paths. The A* algorithm is well-established in the field of path search in 2D environments^18^. If it is directly applied to a 3D environment, the problem of exponential rise in computing data and increase in computing time, which leads to slow search efficiency, needs to be improved. Reference^19^ proposes a model-constrained A * -based three-dimensional trajectory planning for unmanned aerial vehicles. By optimizing the cost function of the traditional A * and selecting extension nodes by controlling the value of the coefficient, the search efficiency of the algorithm is improved. Reference^20^ proposes a model-constrained A * -based three-dimensional trajectory planning for unmanned aerial vehicles. By optimizing the cost function of the traditional A * and selecting extension nodes by controlling the value of the coefficient, the search efficiency of the is improved.

Computational intelligence (CI) algorithms can provide solutions to NP-hard problems with many variables. CI algorithms are a group of nature-inspired methods, which have been raised as a solution for these problems. They can address complex real-world scenarios that algorithms. Genetic Algorithm (GA)^21^ is an adaptive global optimization probabilistic search algorithm developed by simulating the genetic and evolutionary processes of organisms in the natural environment. However, the GA algorithm is time-consuming and generally not suitable for real-time planning. Reference^22^ proposes an improved adaptive GA that adaptively adjusts the probabilities of crossover and genetic operators in a nonlinear manner, enabling the generation of more optimal individuals during the evolution process and obtaining the global optimal solution. Simulation results show that the improved adaptive GA enhances the local search capability of the genetic algorithm, improves the planning efficiency, and can accomplish the UAV path planning task. Particle Swarm Optimization (PSO)^23^ is an evolutionary computational method based on group intelligence. The biggest advantage of PSO is its simplicity, fast operation speed, and short convergence time. However, in the face of high-dimensional complex problems, PSOs often encounter the drawbacks of premature convergence and poor convergence performance, which cannot guarantee convergence to the optimal point. In recent years, the grey wolf optimization (GWO) algorithm has been widely used in various fields^24^. The while optimizer (WOA)^25^ is a GWO-based method because of the success of GWO. Reference^26^ proposes a parallel PSO and enhanced sparrow search algorithm (ESSA) for unmanned aerial vehicle path planning. In the ESSA, the random jump of the producer’s position is strengthened to guarantee the global search ability. Ni and Wu et al.^27^ proposes an improved dynamic bioinspired neural network (BINN) to solve the AUV real-time path planning problem. A virtual target is proposed in the path planning method to ensure that the AUV can move to the real target effectively and avoid big-size obstacles automatically. Furthermore, a target attractor concept is introduced to improve the computing efficiency of neural activities. Ni and Yang^28^ studied the heterogeneous AUV cooperative hunting problem and proposed a novel spinal neural system-based approach. The presented algorithms not only accomplishes the search task but also maintains a stable formation without obstacle collisions. These methods provide some new ideas for the study of UAV path planning in this paper.

Real-time and autonomy in complex flight environments are important indicators for measuring different path-planning algorithms. In the above algorithms, the sampling-based path planning algorithms reduce the traversal search space by sampling, sacrificing the optimality of paths in exchange for a shorter computation time. As the size of the environment increases, the number of operation iterations increases dramatically, making it difficult to achieve simultaneous optimization accuracy and optimal paths in 3D complex environments. Node-based path planning algorithms can obtain the optimal path between the start and endpoints. However, as the environment expands, the dimensionality increases and the number of search nodes increases, the computational size of these algorithms will increase dramatically. Intelligent biomimetic algorithms optimize paths in a mutation-like manner, which can better handle unstructured constraints in complex scenarios. However, its variational solving process requires a long iteration period and cannot be adapted to path planning in dynamic environments^29^.

In response to the limitations of the traditional algorithms mentioned above, a new feasible solution is to update the distance information between the UAVs and the obstacles and target points in real-time and feed it back to the UAV, as well as to make real-time adjustments to its flight status and maneuvers^30^. Reinforcement learning (RL)is a branch of machine learning. The UAVs and the flight environment are modeled using Markov Decision Process (MDP), then the UAV chooses the optimal action to maximize the cumulative reward^31^. UAVs can learn through continuous interaction with the environment, using training and learning to help UAVs gradually master the environment, and continuously optimize the state-behavior to obtain the optimal strategy through the feedback (rewards) given by the environment, which is closer to the human learning process.

In the face of the problem that the environment model is unknown and the transfer probability and value function are difficult to determine, the RL algorithm of interactive learning with the environment to obtain the optimal strategy needs to be a model-free RL algorithm. The Q-Learning (QL) algorithm is one of the most commonly used model-free RL algorithms and has been widely applied to solve path-planning problems. Reference^32^ proposes a Dynamic Fast Q-Learning (DFQL) algorithm to solve the path planning problem of USV in partially known marine environments, DFQL algorithm combines Q-Learning with an Artificial Potential Field (APF) to initialize the Q-table and provides USV with a priori knowledge from the environment. Reference^33^ introduces an Improved Q-Learning (IQL) with three modifications. First, add a distance metric to QL to guide the agent toward the target. Second, modify the Q function of QL to overcome dead ends more effectively. Finally, introduce the concept of virtual goal in QL to bypass the dead end. Reference^34^ proposed a multi-strategy Cuckoo search based on RL. Reference^35^ uses potential field information to simply initialize the Q-value table, giving it certain basic guidance for the target point. Reference^36^ proposes a QL algorithm based on neural networks, which uses Radial Basis Function (RBF) networks to approximate the action value function of the QL algorithm.

All in all, RL takes rewards from exploring the environment as training data by imitating the learning process of human beings and trains itself without requiring preset training data. The path planning algorithm of UAV based on RL senses the state information of obstacles in the environment continuously and inputs the information into the algorithm, The optimal collision-free path can be obtained by adjusting the flight state of the UAV through RL, which can solve the problems of poor real-time and long planning time of traditional trajectory planning.

However, in practice, due to the complexity of the flight environment, the traditional RL algorithms do not run well in complex scenarios. More concretely, the memory size of a Q-table increases exponentially as the dimensionality of the state space or action space associated with the environment increases^37^; The slow convergence caused by dimension explosion will lead to disastrous consequences in path planning, thus limiting the performance of RL in practice; The sparse reward function of the traditional RL algorithm will lead to algorithm convergence difficulties, resulting in the model stops learning and cannot improve; The algorithm faces the "exploration–exploitation dilemma" because it needs to consider both exploration and exploitation in action selection^38^. Therefore, the RL algorithm needs to be improved and optimized before it is used to solve the UAV path planning problem.

## UAV anti-collision control strategy

### UAV spherical safety envelope

**Figure 2:**
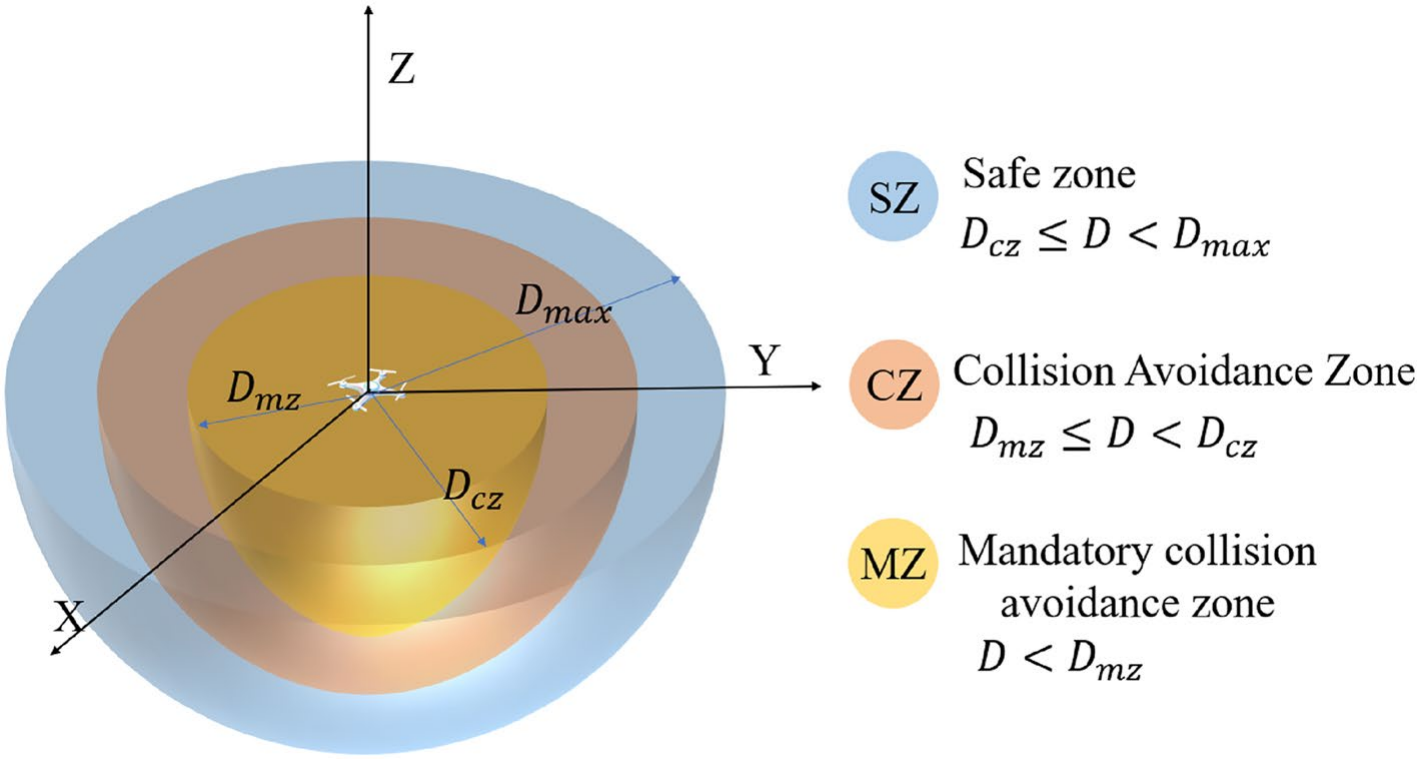
UAV spherical safety envelope profile.

UAVs are unable to obtain complete priori information about the environment during the flight, and can only obtain the information within a certain range centered on themselves through various onboard sensors such as Light Detection And Ranging (LiDAR), and vision sensors. The maximum distance that the sensors of a UAV can detect is defined as ${D}_{max}$. This paper constructs a spherical safety envelope for UAVs The spherical security envelope is centered at the centroid position of the UAV, which is the demarcation of the threat that the UAV can avoid. It can be used to calculate the action reward of the UAV during RL, and act as an event-triggered mechanism for mandatory UAV anti-collision avoidance strategies. As is shown in Fig. 2, the thresholds of the safety zone named SZ) is ${D}_{max}$; the thresholds of the collision avoidance zone (named CZ) and the mandatory collision avoidance zone (named MZ) are represented by ${D}_{cz}$ and ${D}_{mz}$, respectively. When the obstacle is in SZ, there is no collision risk between the UAV and the obstacles; when the obstacle is in CZ, the UAV needs to conduct a collision warning and be aware that the obstacle may enter the MZ; when the obstacle is in MZ, the anti-collision avoidance strategy will be triggered to ensure safety.

### UAV anti-collision avoidance strategies

Before applying APPA-3D to solve the UAV path planning problem, an anti-collision avoidance strategy should be designed. The purpose is to adjust the UAV's flight status such as direction or speed in response to dynamic obstacles (such as other vehicles in the airspace, birds, etc.) to achieve obstacle avoidance. The design of the anti-collision avoidance strategy refers to the method of setting up collision zones in (NMAC) and (COLREGS).

**Figure 3:**
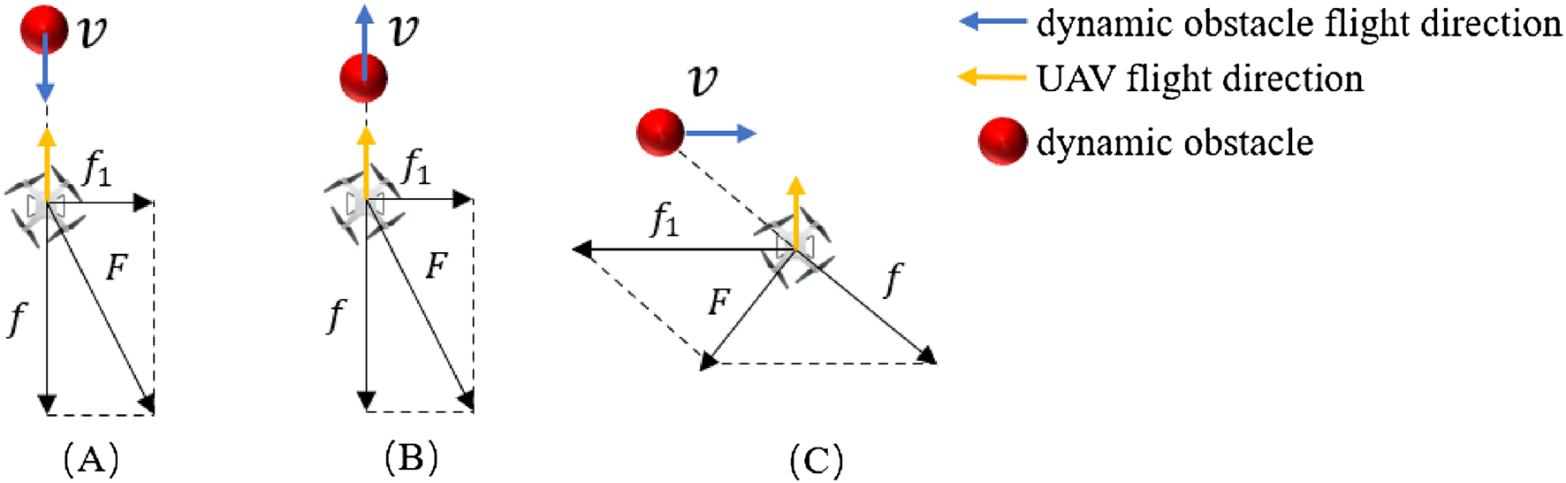
The relative position of the UAV and the dynamic obstacle.

When a dynamic obstacle enters a UAV's MZ, a collision avoidance strategy will be triggered to reduce the risk of collision until the distance between the UAV and the obstacle is greater than ${D}_{mz}$. According to the rules of NMAC, we divide the possible conflict scenarios into flight path opposing conflict、pursuit conflict, and cross conflict. The relative position of the UAV to the dynamic obstacle is shown in Fig. 3.

In Fig. 3, when the flight path of the dynamic obstacle is in the same straight line as the UAV, the two are about to have an opposing conflict or pursuit conflict, and their relative positions are schematically shown in Fig. 3A,B. The vertical direction vector ${f}_{1}$ is added to the direction vector $f$ of the connection between the UAV and the obstacle. The UAV flies along the direction of merging vector $F$ of vectors $f$ and ${f}_{1}$ until it avoids or overtakes an obstacle.

When the flight path of the dynamic obstacle is in the same straight line as the UAV, the two flight paths cross and conflict, and their relative positions are shown in Fig. 3C. Vector $F$ is the combined vector direction of vector $f$ and vector ${f}_{1}$. Vector ${f}_{1}$ is opposite to the direction of obstacle movement $v$. The UAV flies along the merging vector $F$ to avoid the obstacle.

**Figure 4:**
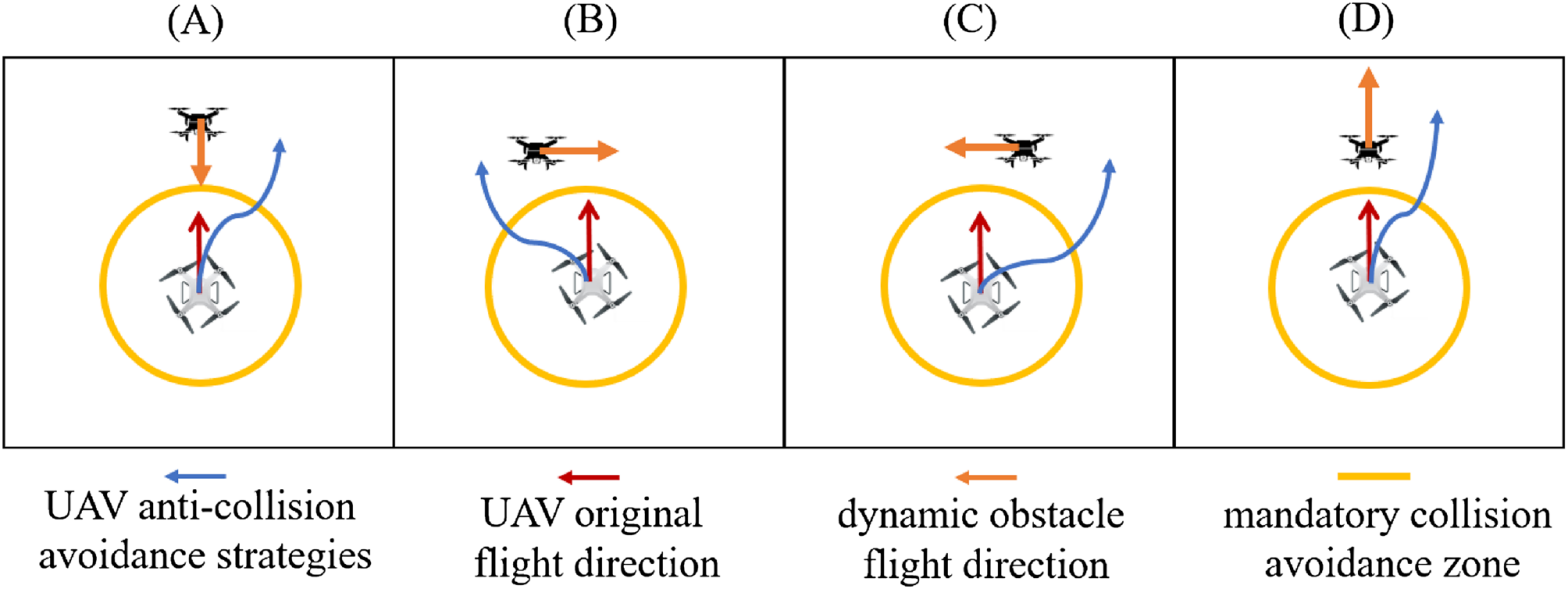
Schematic of UAV anti-collision avoidance strategies.

Based on the different collision scenarios generated by the relative positions of the UAV and dynamic obstacles, four corresponding anti-collision avoidance strategies are designed, as shown in Fig. 4.

In Fig. 4A, there is a risk of opposing conflict between the dynamic obstacle and the UAV. Similar to the method shown in Fig. 3A, the UAV will fly along the merging vector $\overrightarrow{F}$, to avoid obstacles. The flight paths of the UAV and the dynamic obstacle are shown in Fig. 4A.

In Fig. 4B,C, there is a risk of cross-conflict between dynamic obstacles and UAVs. Similar to the method shown in Fig. 3C, the UAV will fly along the merging vector direction $\overrightarrow{F}$ and pass behind the moving direction of the dynamic obstacle, thus the UAV can avoid the dynamic obstacle successfully with the shortest avoidance path. The flight paths of the UAV and the dynamic obstacle are shown in Fig. 4B,C.

In Fig. 4D, dynamic obstacles are in the UAV's path of travel and moving at a speed less than the UAV. There is a risk of pursuit and conflict between the dynamic obstacles and the UAV. Similar to the method shown in Fig. 3B, the UAV will fly along the merging vector direction $\overrightarrow{F}$ to complete the overtaking of the dynamic obstacle. The flight paths of the UAV and the dynamic obstacle are shown in Fig. 4D.

## Design of autonomous collision-free path planning algorithm for UAVs

**Figure 5:**
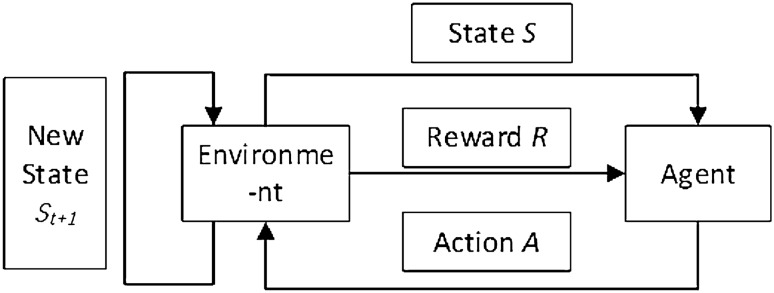
Framework of RL.

The basic framework of RL is shown in Fig. 5.

Essentially, RL is the use of the Agent to interact with the environment constantly, and obtain the optimal value function ${q}^{*}$ for state $S$, through the feedback (reward) given by the environment to continuously optimize the state-action to obtain the optimal strategy ${\pi }^{*}$. The mathematical formula is expressed as Eq. (1) and Eq. (2):

1 $$q^{*} \left( {s,\;a} \right) = \mathop {\max }\limits_{\pi } q_{\pi } \left( {s,\;a} \right)$$

2 $$\pi^{*} \left( {a|s} \right) = \left\{ {\begin{array}{*{20}c} {1, \;\;\;\;\;\;\;\; a = argmax_{a \in A} q^{*} \left( {s,a} \right)}\\ {0, \;\;\;\;\;\;\;\;\;\; other} \\ \end{array} } \right.$$

Thus, the problem of finding the optimal strategy translates into finding the largest of the action state value functions produced under all strategies.

RL-based path planning algorithm allows UAVs to learn and gain rewards through constant interaction with the surroundings through trial and error with little knowledge of the environment and is, therefore, suitable for UAV ‘s path planning under complex conditions. The advantage of an RL-based path planning algorithm is that it can realize path planning in the absence of a priori information about the environment and is highly searchable, but it suffers from the problem of reward sparsity^39^, which can cause convergence difficulties in high-dimensional spaces.

APPA-3D first combines the principle of the APF method and designs an adaptive reward function. Dynamic rewards are generated in real time by judging the effectiveness of UAV movements with environmental information. Secondly, to address the "exploration-utilization dilemma" of RL in the UAV path planning process, an RL action exploration strategy based on action selection probability is proposed. The strategy dynamically adjusts the action selection strategy by combining the size of the value function in different states, to solve the exploration-utilization problem of RL and improve the efficiency of path search.

### Virtual force generation for UAV based on APF

The basic idea of path planning with the APF^40^ is to design the motion of an object in its surroundings as the motion of an abstract artificial gravitational field. The target point has "gravitational force" on the object, while the obstacle has "repulsive force" on the object, and the motion of the object is controlled by the net force.

The current position of the UAV is denoted as $X=\left(x,y,z\right)$; the position of the target point is denoted as ${X}_{g}=\left({x}_{g},{y}_{g},{z}_{g}\right)$; and the position of the start point is denoted as ${X}_{0}=\left({x}_{0},{y}_{0},{z}_{0}\right)$. The gravitational potential field function is defined as Eq. (3):

3 $$U_{att} = \frac{1}{2}kD_{goal}^{2}$$

In Eq. (3), $k$> 0 is the gravitational potential field function coefficient constant. The distance from the UAV to the target point is ${D}_{goal}=\Vert X-{X}_{g}\Vert$, and the gravitational force is the negative gradient of the gravitational potential field function, defined as Eq. (4):

4 $$F_{att} \left( X \right) = - {\triangledown }\left( {U_{att} \left( X \right)} \right) = k\left( {X_{g} - X} \right)$$

Define the repulsive potential field function as Eq. (5):

5 $$U_{rep} \left( X \right) = \left\{ {\begin{array}{*{20}c} {\frac{1}{2}m\left( {\frac{1}{{D_{barrier} }} - \frac{1}{{\rho_{0} }}} \right)^{2} } & {D_{goal} \le \rho_{0} } \\ 0 & {D_{goal} > \rho_{0} } \\ \end{array} } \right.$$

In Eq. (5), $m$> 0 is a repulsive potential field coefficient constant. The position of the obstacle is ${X}_{b}=\left({x}_{b},{y}_{b},{z}_{b}\right)$. The distance from the UAV to the obstacle is ${D}_{barrier}=\Vert X-{X}_{b}\Vert$. ${\rho }_{0}$ is the maximum range of influence of the obstacle. Define the repulsive force as Eq. (6) and Eq. (7):

6 $$F_{rep} \left( X \right) = - {\triangledown }\left( {U_{rep} \left( X \right)} \right) = \left\{ {\begin{array}{*{20}c} {m\left( {\frac{1}{{D_{barrier} }} - \frac{1}{{\rho_{0} }}} \right)\frac{1}{{D_{battier}^{2} }}\frac{{\partial D_{barrier} }}{\partial X}} & {D_{goal} \le \rho_{0} } \\ 0 & {D_{goal} > \rho_{0} } \\ \end{array} } \right.$$

7 $$\frac{{\partial D_{barrier} }}{\partial X} = \left( {\frac{{\partial D_{barrier} }}{\partial x},\;\frac{{\partial D_{barrier} }}{\partial y},\;\frac{{\partial D_{barrier} }}{\partial z}} \right)$$

Thus the net force $F\left(X\right)$ on the UAV is shown in Eq. (8)

8 $$F\left( X \right) = F_{att} \left( X \right) + F_{rep} \left( X \right)$$

### Design of adaptive reward function

The reward function is used to evaluate the actions of the Agent. In traditional RL algorithms, the Agent can only obtain the positive and negative sparse reward function by reaching the target point or colliding with an obstacle. The model does not receive any feedback until it receives the first reward, which may cause the model to stop learning and fail to improve. This reward function will make the algorithm convergence difficult, and in most states cannot reflect the good or bad of its action choice. he sparse reward function $R$ is shown in Eq. (9):

9 $$R = \left\{ {\begin{array}{*{20}c} { - 1,} & {s_{t} = Filure} \\ { + 1,} & {s_{t} = Success} \\ {0,} & {s_{t} = Other} \\ \end{array} } \right.$$

In Eq. (9), ${s}_{t}=Filure$ means that the UAV collides with an obstacle in state t and receives a negative reward − 1, while ${s}_{t}=Success$ means that the UAV reaches the target point in state t and receives a positive reward + 1. Other states have no reward.

To solve the difficult problem of sparse rewards, in this paper, combined with the artificial potential field algorithm, the gravitational force generated by the target point and the repulsive force generated by the obstacle on the agent are converted into the reward or punishment obtained by the agent after performing the action ${a}_{t}$ in the state ${s}_{t}$. The optimized reward function is shown in Eq. (10):

10 $$R = \left\{ {\begin{array}{*{20}c} {R_{a} } & {D \ge D_{cz} { }or{ }D_{cz} \le D < D_{max} } \\ {R_{cz} } & {D_{mz} \le D < D_{cz} } \\ {R_{mz} } & {D < D_{mz} } \\ \end{array} } \right.$$

In Eq. (10), ${R}_{a}$ represents the reward function when the obstacle is within the SZ or when no obstacle is detected. the collision avoidance action reward function is ${R}_{ca}$, and the mandatory collision avoidance action reward function is ${R}_{mz}$.

The Euclidean distance between the starting point of the agent and the target is denoted by ${d}_{max}$, and the Euclidean distance between the current position of the agent and the target is denoted by ${d}_{goal}$. The formula as Eq. (11) and Eq. (12):

11 $$d_{max} = \parallel X_{0} - X_{g}\parallel$$

12 $$d_{goal} = \parallel X - X_{g} \parallel$$

The hyperbolic tangent function can map all will any real number to (− 1, 1). The hyperbolic tangent function can be written as Eq. (13):

13 $$\tanh x = \frac{\sinh x}{{\cosh x}} = \frac{{e^{x} - e^{ - x} }}{{e^{x} + e^{ - x} }}$$

**Figure 6:**
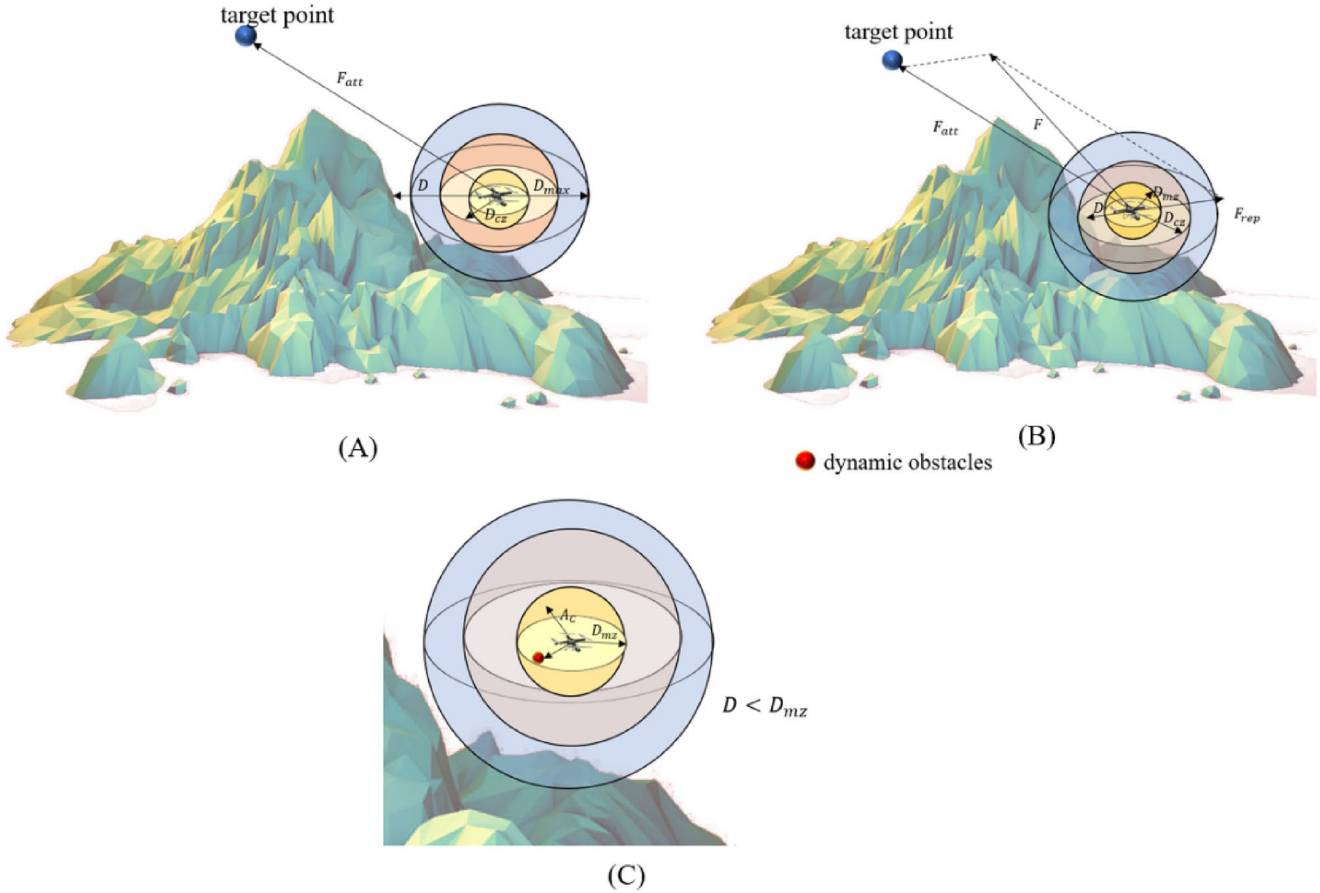
Direction of UAV movement when the obstacle in different zones.

As shown in Fig. 6A, when the obstacle is in the SZ, or no obstacle is detected, the UAV is only affected by the gravitational force ${F}_{att}$ generated by the target point, reward function ${R}_{a}$ is as shown in Eq. (14)

14 $$R_{a} = \left| {\tanh \left( {d_{max} - d_{goal}^{t} } \right)} \right|\frac{{d_{goal}^{t} - d_{goal}^{t + 1} }}{{\left| {d_{goal}^{t} - d_{goal}^{t + 1} } \right|}}$$

In Eq. (14): ${d}_{goal}^{t}$ denotes the Euclidean distance between the agent position and the target point position at moment $t$.

From Eq. (14), it can be seen that after each state change of the agent, if the distance between the agent and the target point under $t+1$ decreases compared to $t$ moments, then ${R}_{a}>0$, the agent gets a positive reward at this time, and vice versa, it gets a negative reward, which is consistent with the principle of RL.

As shown in Fig. 6B, when the obstacle is in the CZ, the UAV is affected by the repulsive force ${F}_{rep}$ of the obstacle and the attractive force ${F}_{att}$ of the target point. At this time, the reward function decreases with the increase of the distance between the agent and the obstacle. The reward function ${R}_{ca}$ can be written as Eq. (15):

15 $$R_{cz} = \left| {\tanh \left( {D_{cz} - d_{barrier}^{t} } \right)} \right|\frac{{d_{barrier}^{t + 1} - d_{barrier}^{t} }}{{\left| {d_{barrier}^{t + 1} - d_{barrier}^{t} } \right|}} + \left| {\tanh \left( {d_{max} - d_{goal}^{t} } \right)} \right|\frac{{d_{goal}^{t} - d_{goal}^{t + 1} }}{{\left| {d_{goal}^{t} - d_{goal}^{t + 1} } \right|}}$$

where: ${d}_{barrier}^{t}$ denotes the Euclidean distance between the agent position and the obstacle position at moment $t$.

From Eq. (15), it can be seen that the reward function ${R}_{ca}$ when the obstacle is in the CZ consists of two parts, one is the reward generated by the obstacle to the UAV, if the distance between the UAV and the obstacle under the $t+1$ moment is reduced compared to the $t$ moment, then the reward generated by the obstacle to the agent is negative, and vice versa is positive. The other is the reward generated by the target point to the UAV, and the principle is the same as Eq. (5). When the obstacle is in the CZ, the agent accepts the reward function of the obstacle and the target point at the same time, which can solve the defects that the traditional APF method is easy to fall into the local minima and oscillate in the narrow passage, to guide the UAV out of the trap area and move toward the target point smoothly.

As shown in Fig. 6C, when the obstacle is within the MZ, the risk of UAV collision with the obstacle is high. To prevent conflicts, A collision avoidance strategy ${A}_{C}$ is mandatory for the UAV, The reward function ${R}_{ca}$ can be written as Eq. (16):

16 $$R_{mz} = \left| {\tanh \left( {D_{cz} - d_{barrier}^{t} } \right)} \right|\frac{{d_{barrier}^{t + 1} - d_{barrier}^{t} }}{{\left| {d_{barrier}^{t + 1} - d_{barrier}^{t} } \right|}}$$

The adaptive reward function is consistent with RL. By converting the reward values of each action-state into continuous value between (− 1, 1), the problem of sparse reward functions is solved. The adaptive reward function solves the problem that traditional reward functions can only earn positive or negative rewards by reaching a target point or colliding with an obstacle, and other actions do not receive any positive or negative feedback. The adaptive reward functions are generated by determining the validity of the executed action and environmental information. Compared to the traditional sparse reward function, the adaptive reward function proposed in this paper combines the good performance of APF to make the reward accumulation process smoother, and can also reflect the relationship between the current state of the UAV and the target state.

### Action exploration strategy optimization of reinforcement learning

In the process of constant interaction with the environment, the Agent keeps exploring different states and obtains feedback on different actions. Exploration helps the Agent to obtain feedback through continuous experimentation, and Exploitation is where the Agent refers to the use of existing feedback to choose the best action.

On the one hand, RL obtains more information by exploring more of the unknown action space to search for the global optimal solution, but a large amount of exploration reduces the performance of the algorithm and leads to the phenomenon of non-convergence of the algorithm. On the other hand, too much exploitation will fail to choose the optimal behavior because of the unknown knowledge of the environment. Therefore how to balance exploration and utilization is an important issue for the Agent to continuously learn in interaction.

There is a contradiction between "exploration" and "exploitation ", as the number of attempts is limited, and strengthening one naturally weakens the other. Excessive exploration of the unknown action space can degrade the performance of the algorithm and lead to non-convergence of the algorithm while obtaining more information to search for a globally optimal solution. In contrast, too much exploitation prevents the selection of optimal behavior because of the unknown knowledge of the environment. This is the Exploration—Exploitation dilemma faced by RL. To maximize the accumulation of rewards, a better compromise between exploration and exploitation must be reached.

Action exploration strategies can be categorized into directed and undirected exploration methods. The directed exploration approach reduces the blindness in the pre-exploration phase of action exploration and thus improves the exploration efficiency by introducing a priori knowledge into the action exploration strategy. directed exploration methods, on the other hand, make a compromise between exploration and exploitation by setting parameters, and the usual approaches are the $\epsilon -greedy$ strategy and the Softmax distribution strategy.

The $\epsilon -greedy$ strategy usually sets a parameter $\epsilon$ to select the current optimal action with a probability of $\left(1-\epsilon \right)$, and randomly selects among all the actions with a probability of $\epsilon$, which is represented by Eq. (17):

17 $$a = \left\{ {\begin{array}{*{20}l} {Actions \;selected \;with \;uniform \;probability\; in\; A} \hfill & {P = \varepsilon } \hfill \\ {a = argmax_{a \in A} Q\left( {s,\;a} \right)} \hfill & {P = 1 - \varepsilon } \hfill \\ \end{array} } \right.$$

In Eq. (17), When $\varepsilon$ is 0, the $\epsilon -greedy$ strategy is transformed into a greedy strategy, and the degree of exploration gradually increases as $\varepsilon$ is gradually increased from 0 to 1; when $\varepsilon$ is 1, the $\epsilon -greedy$ strategy is transformed into a randomized choice action. Although the $\epsilon -greedy$ strategy solves the problem between exploration and exploitation to a certain extent, the problem of exploration and exploitation still exists because the parameter $\epsilon$ is fixed and there are problems such as the difficulty of setting the parameter $\epsilon$, and the lack of differentiation between non-optimal actions.

The Softmax distribution strategy makes a tradeoff between exploration and exploitation based on the average reward of currently known actions. If the average rewards of the maneuvers are comparable, the probability of selecting each maneuver is also comparable; if the average reward of some maneuvers is significantly higher than that of other maneuvers, the probability of their selection is also significantly higher.

The action assignment for the Softmax distribution strategy is based on the Boltzmann distribution, which is represented by Eq. (18):

18 $$P\left( {a_{i} |s} \right) = \frac{{e^{{\frac{{Q\left( {s,\;a_{i} } \right)}}{\tau }}} }}{{\mathop \sum \nolimits_{j = 1}^{m} e^{{\frac{{Q\left( {s,\;a_{j} } \right)}}{\tau }}} }}$$

In Eq. (18), $Q\left(s,{a}_{j}\right)$ records the average reward of the current action; $\tau >0$ is called “temperature”, The smaller of $\tau$, the higher the probability of selecting actions with higher average rewards. When τ tends to 0, Softmax tends to " exploitation only", when τ tends to infinity. Softmax tends to "exploration only".

Both $\epsilon -greedy$ strategy and the Softmax distribution strategy are iterated in such a way that the action with the largest action-value function has the largest probability of selection. Based on this, this paper proposes a new action selection strategy, the new strategy solves the balance problem between exploration and exploitation by introducing the concept of "action selection probability " and making action preference selection accordingly.

Action selection probability represents the probability value that an Agent chooses to perform an action in a given state. As shown in Eq. (19), the initial value of the action selection probability for a state-action is the inverse of the size of the action set for that state:

19 $$P\left( {a|s} \right) = \frac{1}{{card\left( {A_{s} } \right)}}$$

In Eq. (19), $card\left({A}_{s}\right)$ denotes the number of actions in the action set ${A}_{s}$ in state $s$.

The action selection probability is dynamically adjusted as the size of the value function of the action changes. During the RL process, Agent in state $s$, selects action $A$ based on the size of the action selection probability value, and after executing the action, Agent obtains the reward $R$ and enters state ${S}^{\mathrm{^{\prime}}}$ and selects the action ${A}^{\mathrm{^{\prime}}}$ with the largest value function to update the value function. Subsequently, the value function for each action in state $s$ is is divided into two parts according to the size of the value: The largest value function is the first part; the rest is the second part. Reduce the probability values of each action in the second part by half and add them evenly to the first part.

The Agent updates the action selection probability after completing an action, according to the size of the state action value function. The update rule is as Eq. (20):

20 $$\left\{ {\begin{array}{*{20}l} {P\left( {a_{i} |s} \right) + \mathop \sum \limits_{{j = 1{}a_{j} \notin A^{*} \left( s \right)}}^{n} \frac{{P\left( {a_{j} |s} \right)}}{m}} \hfill & {a_{i} \in A^{*} \left( s \right) = \arg \max_{a \in A} Q^{*} \left( {s,\;a} \right)} \hfill \\ {P\left( {a_{j} |s} \right)\left( {1 - \frac{1}{m}} \right)} \hfill & {other} \hfill \\ \end{array} } \right.$$

In Eq. (20): $m$ is the rate of change, which represents the rate of change of the action probability; ${A}^{*}\left(s\right)$ is the set of actions with the largest value function, ${a}_{i}$ is the action of the set ${A}^{*}\left(s\right)$, and ${a}_{j}$ is the action with non-maximum value function.

In the initial phase of the algorithm, each action has the same probability of being selected by the Agent, i.e., the action selection probabilities $P\left(a|s\right)$ are equal, at which point the Agent will randomly select the action.

After an Agent completes the exploration of an action, if this exploration results in $R<0$, the action selection probability for that action is halved, at which point the probability of other actions being selected increases, so that in the early stages of the exploration the Agent will be more inclined to select actions that have not been performed. If $R>0$ for this exploration, it indicates that this exploration is a beneficial exploration, which will increase the action selection probability of this action, when the probability of other actions being selected decreases, and therefore the Agent tends to select this action more often; However, there is still a probability of exploration for other actions, thus reducing the risk of action exploration falling into a local optimum.

The pseudo-code for APPA-3D is shown in Algorithm 1:

**Algorithm 1:**
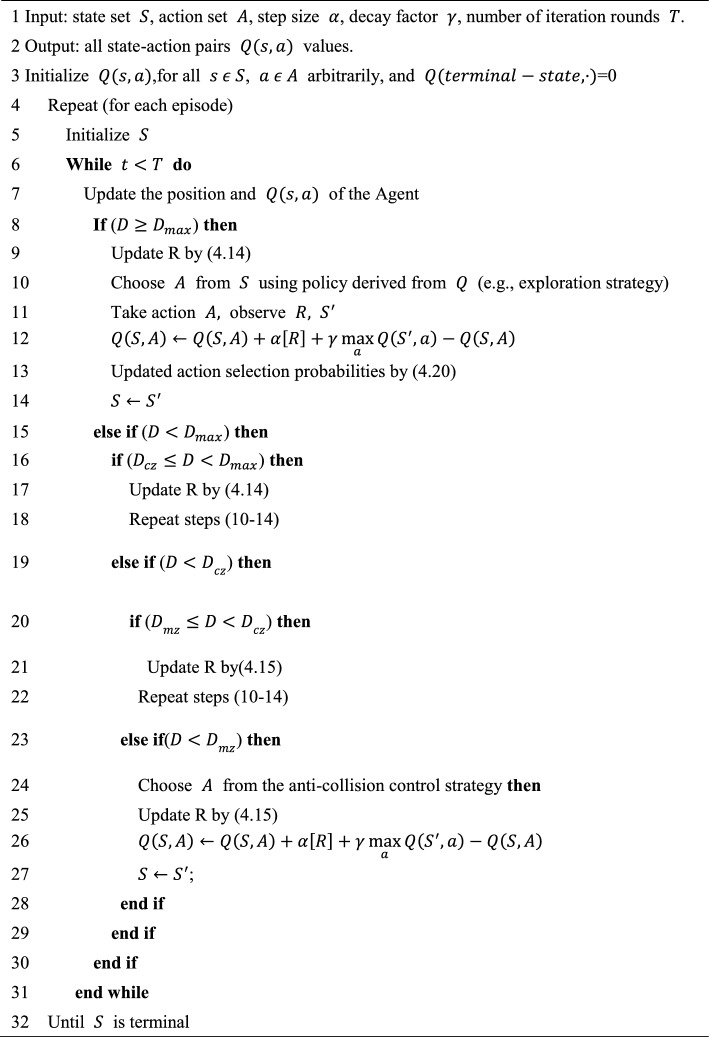
APPA-3D.

## Experiment and results

To verify the feasibility of an autonomous path planning algorithm in complex 3D environments (APPA-3D) for UAVs, this paper selects real environment maps to conduct simulation experiments. The UAV's range of action is limited to the map, and if the UAV moves outside the range of the map or above the low altitude limit altitude, it is determined that a collision has occurred. The starting point for UAV path planning is represented by a black dot, and the target point is represented by a red dot. The maximum flight altitude is 1 km above the peak line, and the no-fly zone is indicated by a green cylinder. The UAV needs to avoid mountains and no-fly zones to fly from the starting point to the target point.

### UAV anti-collision avoidance strategies experiments

**Figure 7:**
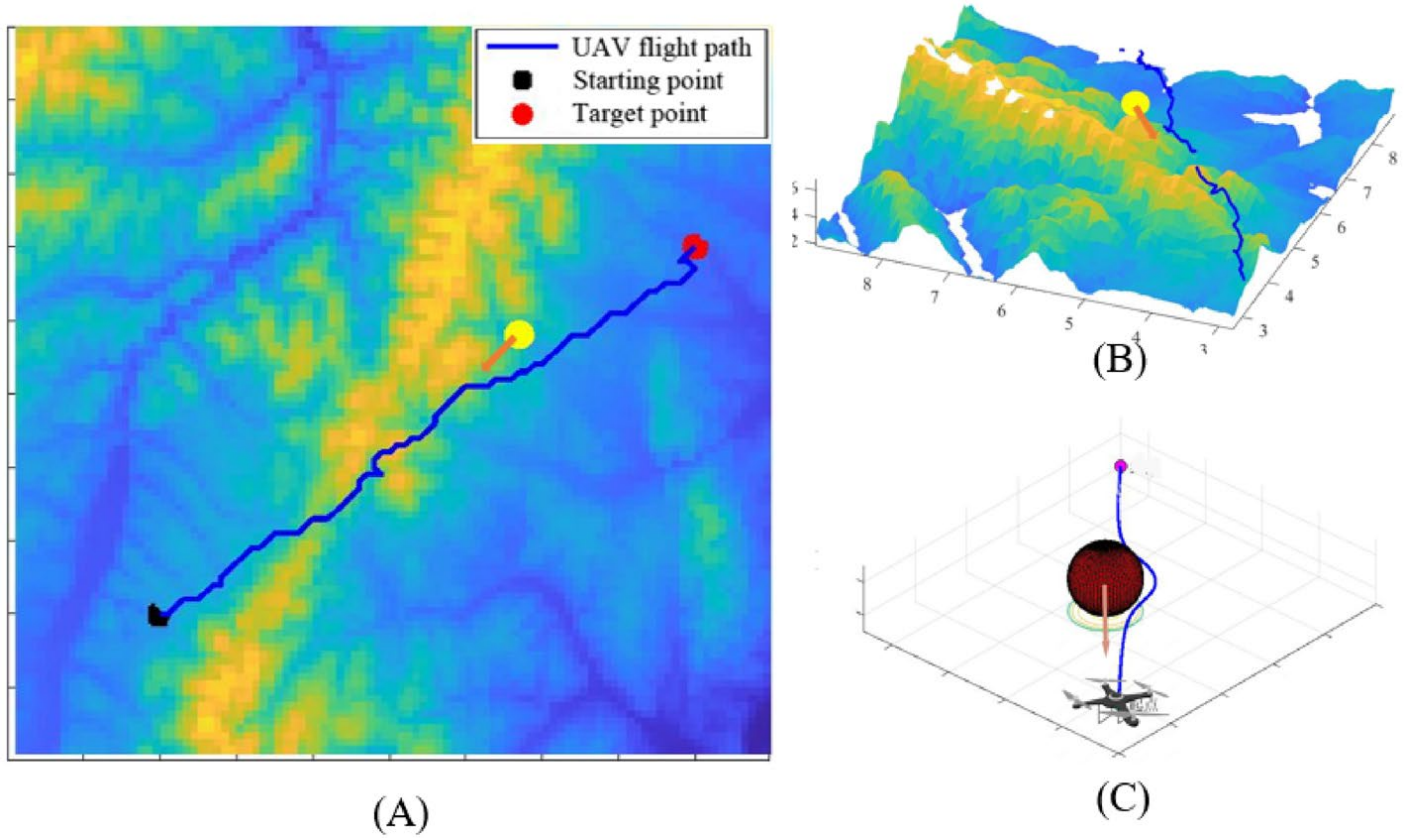
Opposing Conflict Avoidance Simulation.

**Figure 8:**
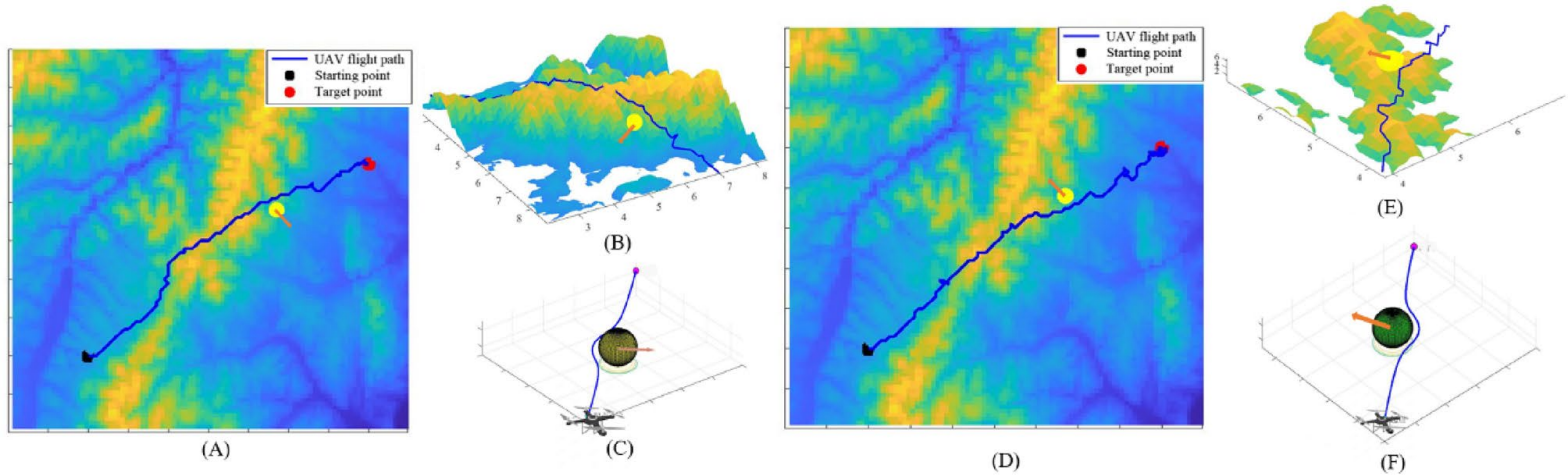
Cross-conflict avoidance simulation.

**Figure 9:**
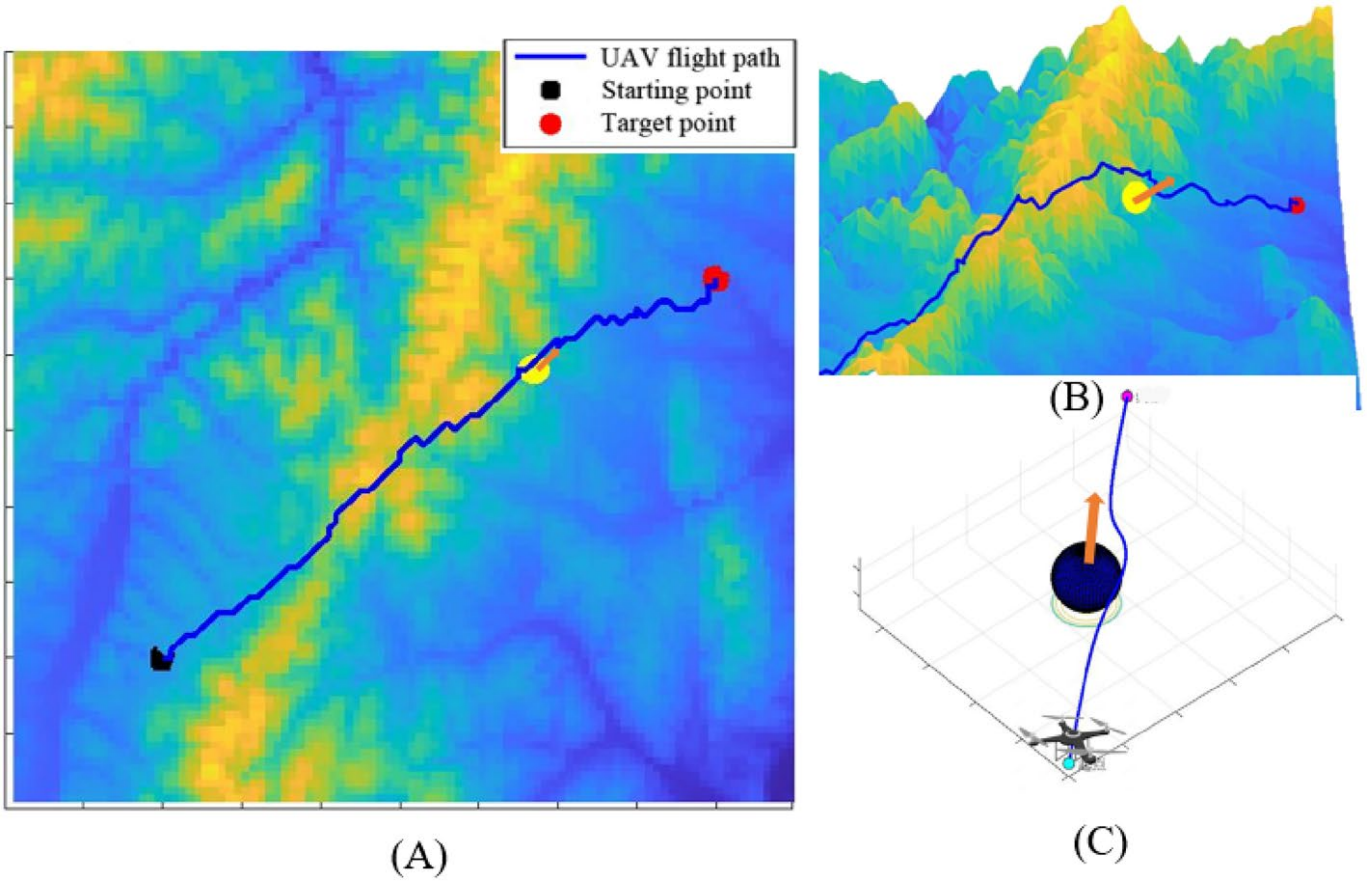
Pursuit conflict avoidance simulation.

The anti-collision avoidance strategies experiments were designed to verify whether APPA-3D can achieve anti-collision avoidance strategies while implementing path planning. Figures 7, 8 and 9 are simulation experimental diagrams of anti-collision avoidance strategies for UAVs.

As shown in Fig. 7, in the opposing conflict avoidance simulation, the intrusion direction of the dynamic obstacle is set to be directly opposite the movement direction of the UAV. When a dynamic obstacle is detected, the UAV chooses to turn right to avoid the dynamic obstacle according to the anti-collision avoidance strategy. The reward function is recalculated after finishing the anti-collision avoidance strategy and guiding the UAV to continue flying toward the target point.

As shown in Fig. 8, in the cross-conflict avoidance simulation, dynamic obstacles invade from the left and right sides of the UAV's flight direction. According to the anti-collision avoidance strategy, when the UAV detects a dynamic obstacle and chooses to pass behind the moving direction of the dynamic obstacle, it can ensure that the UAV and the dynamic obstacle are avoided successfully and the avoidance path is the shortest. The reward function is recalculated after finishing the anti-collision avoidance strategy and guiding the UAV to continue flying toward the target point.

As shown in Fig. 9, in the pursuit conflict avoidance simulation, the flight direction of the dynamic obstacle is the same as the UAV flight direction. Referring to the anti-collision avoidance strategy, when the UAV detects a dynamic obstacle, the UAV chooses to complete the overtake from the right side of the dynamic obstacle's direction of motion, which ensures that the UAV can successfully overtake the dynamic obstacle with the shortest overtake avoidance path; The reward function is recalculated after finishing anti-collision avoidance strategy and guiding the UAV to continue flying towards the target point.

### Multi-obstacle path planning and collision avoidance verification

**Figure 10:**
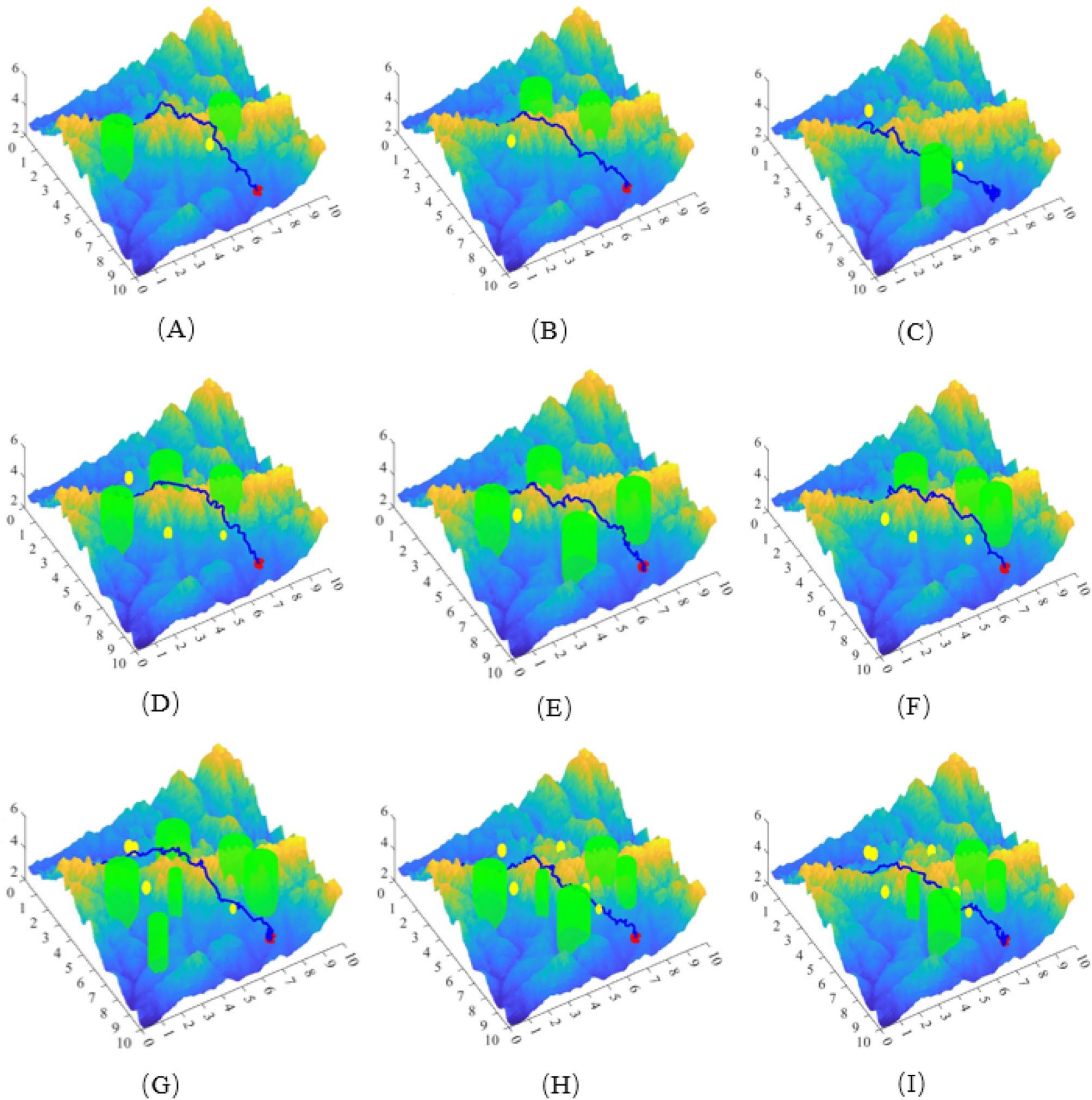
3D view of APPA-3D flight path.

To verify the performance of the APPA-3D, this paper randomly generates 3, 6, and 10 different moving and static obstacles in the same simulation environment and conducts three sets of randomized experiments each. The 3D view of APPA-3D is exhibited in this paper, as shown in Fig. 10.

The 3D view of the paths planned by APPA-3D shows that the flight paths of the UAVs are feasible in nine different scenarios. The distance between the UAV and the obstacle is well-maintained in complex terrain. This further demonstrates that APPA-3D can help the UAV to plan a path that is both short and safe at the same time.

This paper calculates four parameters: UAV path planning time, planned path length, number of planned path points, and planned path ground projection length in 9 scenarios, the average values of the four parameters are shown in Table 1.

**Table 1:** Simulation results in different scenarios.

Number of random obstacles	Path planning time	Planning path length	Number of planning path points	Planning path ground projection length
3	32.30 s	14.31083 km	94	11.51960 km
6	45.05 s	14.28151 km	118	13.87523 km
10	51.12 s	13.74009 km	85	10.29533 km

### Comparative experiments

To verify the enhancement effect of the adaptive reward function and the new action selection strategy proposed in this paper, two sets of ablation experiments were conducted firstly before conducting the comparison experiments.

**Figure11:**
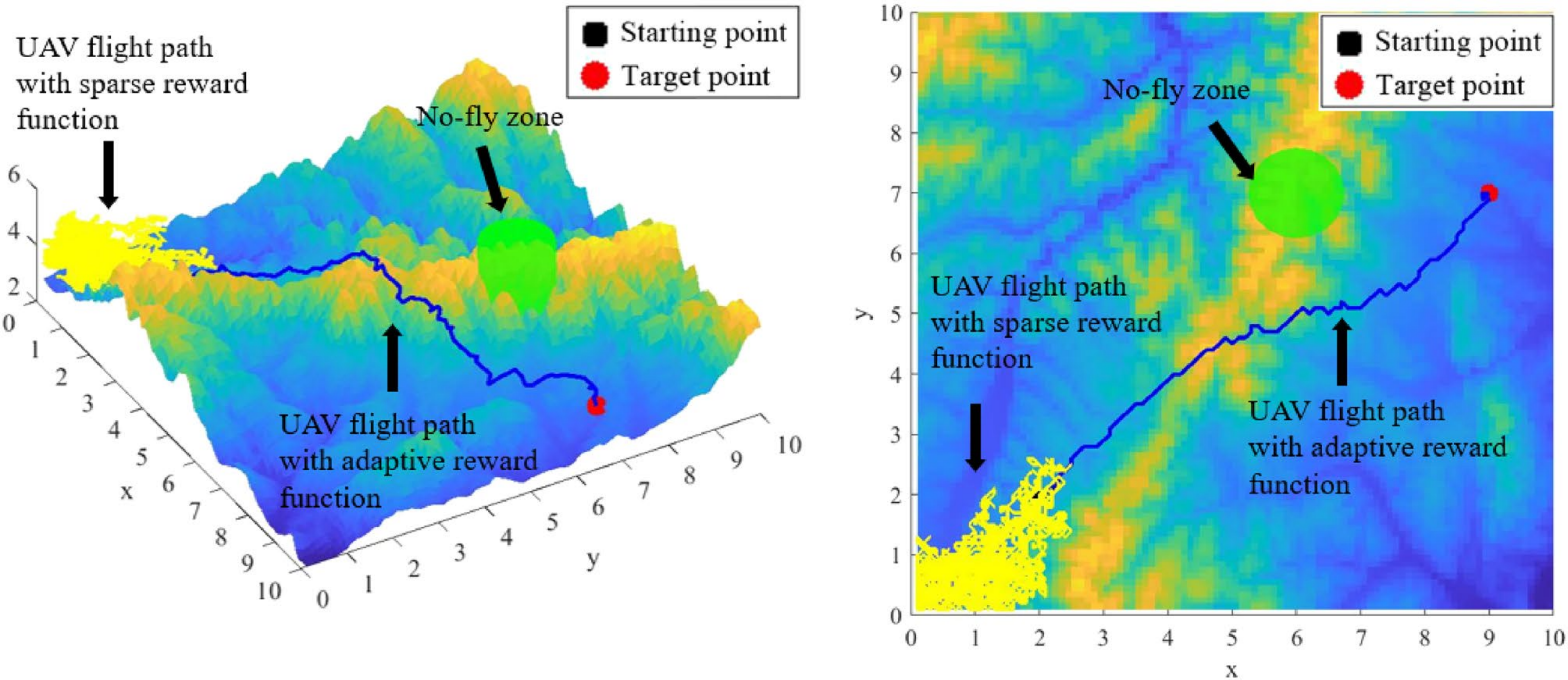
UAV path planning results under sparse reward function and adaptive reward function.

The first set of ablation experiments is to verify the enhancement effect of the new adaptive reward function proposed in this paper, and the experimental results are shown in Fig. 11:

The yellow path in Fig. 11 represents the UAV flight path under the sparse reward function, and the blue path represents the flight path under the adaptive reward function proposed in this paper. Figure 11 clearly shows that the performance of UAV path planning based on sparse reward function is poor in complex 3D environments. This is because under sparse reward function, it can only obtain positive and negative reward when reaching the target point or colliding with obstacles, and other actions will not get any positive or negative feedback. So the UAV is random flight blindly, unable to find the correct flight direction in this way. Compared with the sparse reward function, the adaptive reward function we proposed combines the good performance of APF to make the reward accumulation process smoother and can also reflect the relationship between the current state and the target state of the UAV.

To verify the improvement effect of the new action selection strategy proposed in this paper, the second set of ablation experiments was set up. The experiments were analyzed using the $\epsilon -greedy$ strategy, Softmax distribution strategy, and the new action selection strategy. All RL algorithms adopt Q-learning algorithm, which excludes the influence of learning algorithm on different exploration strategies.

Tables 2 and 3 show the results of three exploration strategies. To prevent the impact of single data on the experiment, the data in Tables 2 and 3 is the average value obtained after 5 experiments.

**Table 2:** Number of planning path points for different strategies.

	$\epsilon -greedy$	Softmax	Action selection strategy
Number of planning path points	112	96	85

**Table 3:** Planning path time for different strategies.

	$\epsilon -greedy$	Softmax	Action selection strategy
Planning path time	68.33 s	60.15 s	53.37 s

The experimental results show that after a period of exploration, three different exploration strategies are able to guide the UAV to the target point. Compared with the other two exploration strategies, the action selection probability we proposed is more advantageous in terms of path planning time and number of path planning points.

To evaluate whether the APPA-3D proposed in this paper has significant advantages over other classical or RL based algorithms, two sets of experiments were utilized to test the ability of the six methods to solve path planning problems under the same conditions. According to the characteristics of algorithms, they can be divided into two categories: classic algorithms (APF, RRT, and A*) and QL-based algorithms (DFQL, IQL, and MEAEO-RL). It should be noted that, to prevent the impact of single data on the experiment, the data in Table 4, 5, 6 and 7 is the average value obtained after 5 experiments.

**Table 4:** Path length of different algorithms.

	A*	RRT	APF	APPA-3D
Planning path length	15.3205 km	16.5351 km	14.9667 km	13.9533 km

**Table 5:** Path planning time of different algorithms.

	A*	RRT	APF	APPA-3D
Planning path time	49.12 s	59.67 s	51.34 s	52.45 s

**Table 6:** Path lengths of four algorithms.

	DFQL	IQL	MEAEO-RL	APPA-3D
Planning path length	15.0154 km	14.8301 km	14.6506 km	13.9533 km

**Table 7:** Path planning time of four algorithms.

	DFQL	IQL	MEAEO-RL	APPA-3D
Planning path time	54.46 s	57.34 s	54.76 s	52.12 s

**Figure12:**
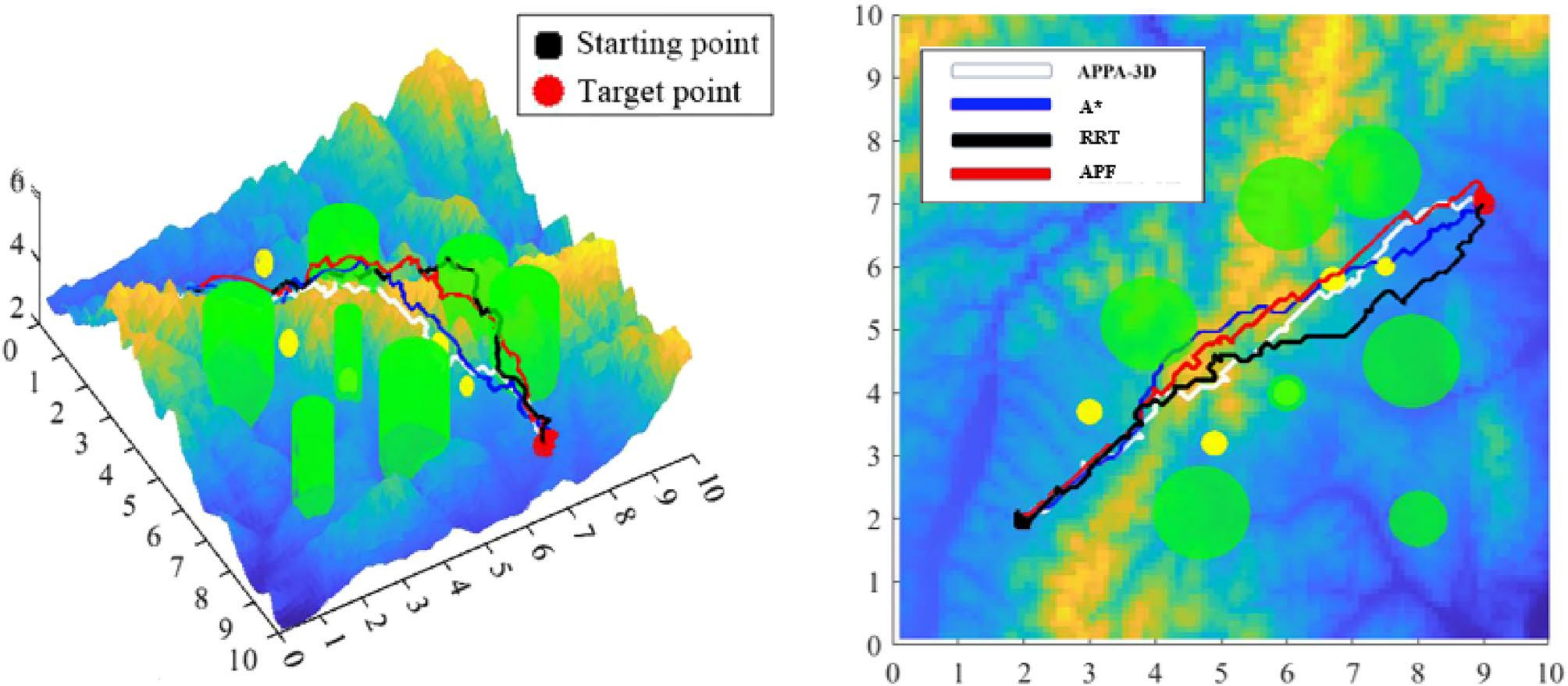
Path planning comparison of A*, RRT, APF and APPA-3D.

The experimental results of the first group are displayed in Fig. 12 and Tables 4 and 5:

It can be seen from Fig. 12 that the three classic algorithms perform better than APPA-3D algorithm in the front part of the path. However, the performance of classic algorithms is poor in the latter part of the path, which is caused by their algorithm characteristics. Because sampling-based and search-based characteristics of algorithms respectively, it is hard to generate smooth and optimal paths with RRT and A*. Although the path planning time of A* is short, the UAV collided with obstacles unfortunately. The reason for the poor effect of APF is that the obstacle surrounds the destination, and the repulsive force field of the obstacle directly acts on the agent, making it unable to approach the obstacle. The agent can only move in the direction where the gravitational force is greater than the repulsive force.

**Figure13:**
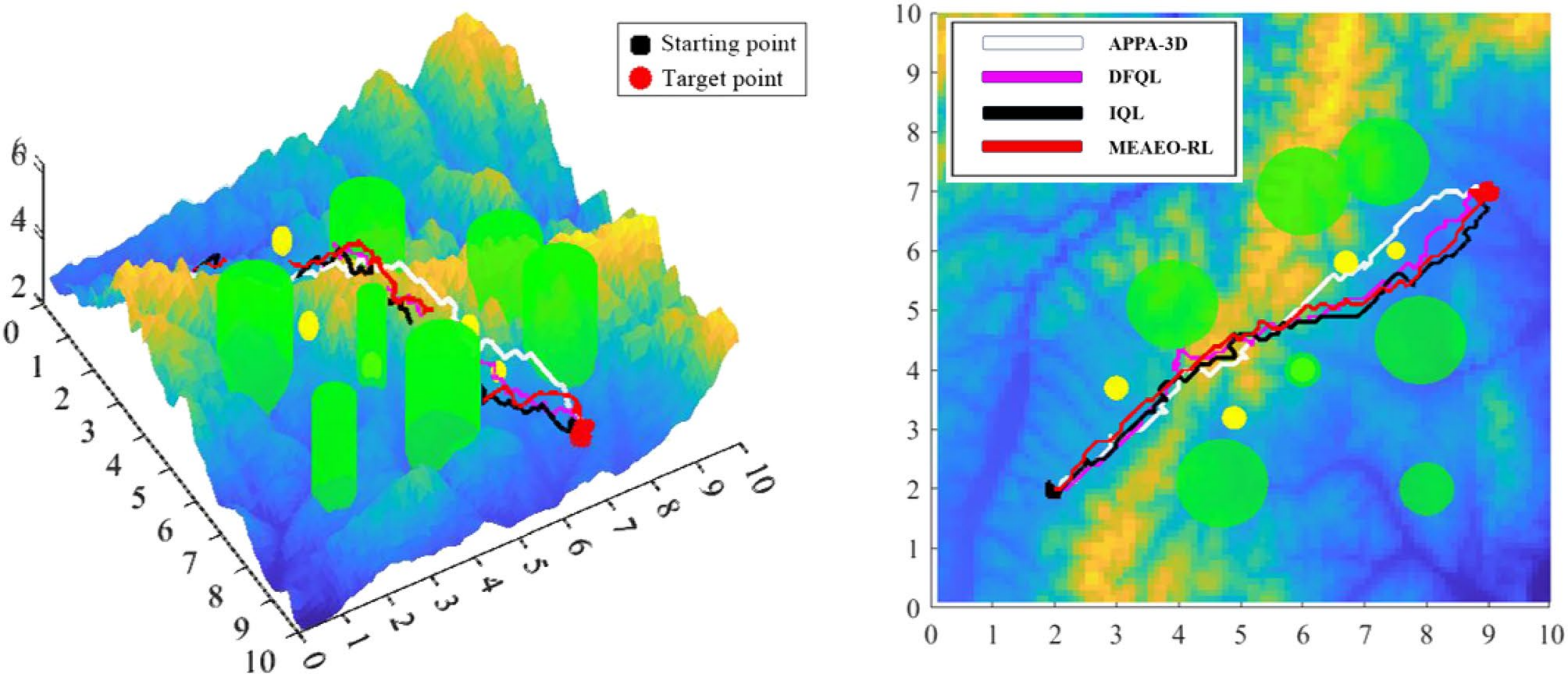
Path planning comparison of DFQL, IQL, MEAEO-RL and APPA-3D.

The results of the second group of experiments are displayed in Fig. 13. It is worth mentioning that DFQL, IQL, MEAEO-R, and APPA-3D are all optimized based on traditional RL algorithms. The simulation results are shown in Fig. 13, Table 6 and Table 7.

Experimental results clearly show that APPA-3D can reach the destination with the shortest distance and time. In the initial phase of path planning, APPA-3D is not very different from other algorithms, and all four algorithms can help the UAV plan a relatively high-quality flight path quickly. While in the middle and later stages of path planning, the differences between APPA-3D and the other three compared algorithms can be seen clearly, especially when facing multi-dynamic obstacles. Because RL algorithm assigns a probability to each possible action and selects the action based on these probabilities, path planning algorithms based on RL often fall into the dilemma of exploration–exploitation when facing complex environments.

To solve this problem, the APPA-3D algorithm proposes a new action selection strategy. This strategy solves the balance problem between exploration and exploitation by introducing the concept of *action selection probability* and making action preference selection accordingly.

**Figure14:**
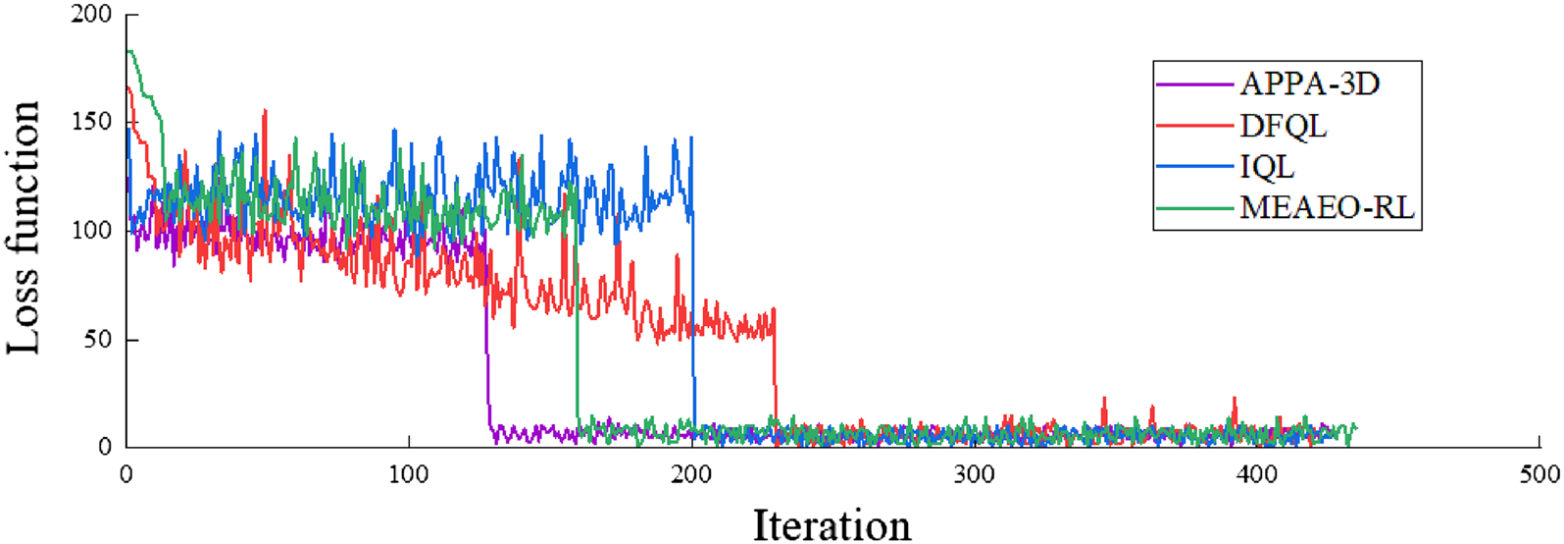
The loss function of DFQL, IQL, MEAEO-RL and APPA-3D.

The Fig. 14 presents the loss function used to observe the convergence behavior over iterations of all algorithms. It can be seen that after about 130 iterations, the loss function begins to stabilize. The rapid convergence of value loss also shows that the APPA-3D is more accurate, which is a good performance and means that the agents won’t fall into a local optimum. The algorithms compared requires more iteration to complete convergence. This is because they use $\epsilon -greedy$ strategy or Softmax distribution strategy as an action exploration strategy of reinforcement learning. And their performance is consistent with the results of the second set of ablation experiments.

In conclusion, APPA-3D is far better than the compared algorithms in the 3D UAV path planning optimization problem. This is because APPA-3D dynamically adjusts the action selection strategy by combining the size of the value function in different states, thus solving the problem of exploration-utilization of RL and improving the efficiency of path search.

## Conclusion

The path planning problem in unknown environments is the focus of UAV task planning research and the key to achieving autonomous flight. Therefore, UAVs need to have the ability to autonomous path planning and avoid potential obstacles. In this paper, an autonomous collision-free path planning algorithm for unknown complex 3D environments is proposed. Firstly, based on the environment sensing capability, a UAV collision safety envelope is designed, and the anti-collision control strategy is investigated, which can effectively deal with the collision problem triggered by dynamic obstacles in the flight environment. Secondly, this paper optimizes the traditional RL algorithm. On the one hand, the reward function for RL is optimized by transforming the relationship between the current state of the UAV and the task into a suitable dynamic reward function. The presence of a dynamic reward function allows the UAV to fly toward the target point without getting too close to the obstacles. On the other hand, an RL action exploration strategy based on action selection probability is proposed. The strategy dynamically adjusts the action selection strategy by combining the size of the value function in different states, thus solving the RL exploration-utilization problem and improving the efficiency of path search. To verify the effectiveness of the designed APPA-3D algorithm in the dynamic collision avoidance model, three typical collision experiments were set up, including flight path opposing collision, pursuit collision, and cross collision. The experimental results verify that the APPA-3D can effectively avoid safety threats that may be caused by dynamic obstacles in complex environments according to the designed anti-collision control strategy. Meanwhile, the results of the algorithm testing experiments in nine different scenarios verified that the algorithm still performs well in the face of random and complex flight environments.

APPA-3D demonstrates better performance in path planning performance comparison tests with other classical and novel optimized RL algorithms. The advantages in path planning length and convergence curves again show that APPA-3D can effectively help UAVs solve the path planning problem.

## Data Availability

The datasets used and analysed during the current study will be available from the corresponding author on reasonable request.
